# Environmental footprints of the data center service sector in Sweden

**DOI:** 10.1016/j.heliyon.2024.e31290

**Published:** 2024-05-18

**Authors:** Kim Jerléus, Muhammad Asim Ibrahim, Anna Augustsson

**Affiliations:** Department of Biology and Environmental Science, Linnaeus University, Sweden

**Keywords:** Data center, Information and communication technology, Electricity usage, Environmental footprints, Sustainable siting of DCs

## Abstract

The global data center (DC) sector has expanded rapidly during the last decades, due to the rising demand for digital services. In the Nordic region, Sweden has emerged as a global hub, attracting leading technology companies like Amazon, Facebook, Microsoft, and Google. Server halls of DCs are energy intensive buildings, which puts pressure on local water resources and contributes to global greenhouse gas emissions. This study aims to, firstly, quantify the environmental impact of DCs, based on energy usage, water consumption, and greenhouse gas (GHG) emissions. Secondly, it develops a planning tool by employing a multi-criteria approach to optimally locate new DCs and to assess the site suitability of existing ones in Sweden. Data of various performance indicators (geographical data on renewable energy accessibility, free cooling conditions, excess heat receivers, and resilience to water shortages) of DCs was collected through different means, e.g., questionnaire surveys, permit applications, company websites, and other open online data repositories. ArcGIS Pro was employed for spatial analysis, and 68 DCs with a site suitability index (SSI) ≤ 45 % were identified as less ideally located. The principal findings are centered on Sweden, and thereby primarily benefit stakeholders engaged in decision-making for evaluating existing or strategic planning of new DCs by incorporating a comprehensive environmental perspective. Given the rapidly changing climate, strategically siting DCs will become crucial for minimizing the sector's environmental impact.

## Nomenclature

CO_2eq_ =: Carbon dioxide equivalents
*DC* =: Data center
*DC_E*_*total*_ =: The annual energy use of a data center (MWh/year)
${DC}_{\text{GHG}}$ =: Annual GHG emissions of a data center
${DC}_{indirect\;H2O}$ =: Annual indirect water consumption of a data center
*DC_P*_*total*_ =: The total power demand of a data center (MW)
${DC}_{direct\;WF}$ =: water consumed directly for cooling purposes
*DHP* =: District Heating Production (GWh/year)
*SSI* =: Environmental Sustainability Index
*E* =: Energy use
*EPp* =: Amount of electricity produced from each primary energy source
*EPt* =: Total electricity produced within each of the four electricity zones
*F*_*p_GHG*_ =: GHG emission factor for a certain primary energy source (kgCO_2eq_/MWh)
*F*_*p_H2O*_ =: Water consumption factor for a certain primary energy source (m^3^/MWh)
${Avg}_{GHG}$ =: Average GHG emission (Kg of CO2.eq/MWh) for a certain electricity zone
${Avg}_{H20}$ =: Average water consumption (m^3^/MWh) for a certain electricity zone
*GWh* =: Gigawatt hour
*GHG* =: Green House Gas
*ICT* =: Information and Communication Technology
*IT*_*d*_ =: Power density of IT equipment (kW/m^2^)
*kWh* =: Kilowatt hour
*LCA* =: Life Cycle Assessment
*MWh* =: Megawatt hour
*NAT* =: Normal Average Ambient Temperature (^o^C)
*P* =: Power (MW)
*p* =: Primary energy source
*P*_*Thermal*_ =: Installed thermal power of a data center back up power plant
*PUE* =: Power Usage Effectiveness
*REU* =: Renewable Energy Utilization (%)
*SE* =: Electricity zone
*TWh* =: Terawatt hour
*UR* =: Utilization Rate
*WEI* =: Water Exploitation Index (%)
*WUE* =: Water Usage Efficiency (m^3^/MWh)
*ZB* =: Zettabyte
*η* =: Thermal efficiency (%)

## 1 Introduction

Data centers (DCs) are vital components of the modern information and communication technology (ICT) infrastructure worldwide, offering a range of services for data storage and management, data backup, validation of transactions, data sharing among users, and data security. The ongoing introduction of new technologies, such as artificial intelligence, edge computing, and the Internet of Things, is driving a significant increase in the demand of data storage and management, with an expected projection of the global data sphere from 33 Zettabytes (ZB) (base year 2018) to 175 ZB by 2025 [1]. According to Yuan et al. [2], the global expansion of the data center sector is particularly pronounced in the Nordic region. This region is favored for establishing new DCs due to its climate compatibility, stable political system, strong economic conditions, and access to cheap and green energy [3,4].

ICT technologies are considered vital for promoting environmental sustainability [5,6], yet DCs, which are essential for the functioning of ICT technologies, have a negative impact on the environment. Data center server halls are extremely energy intensive due to the installation of high-density IT equipment, which consume 10 to 100 times more electricity than that of most other buildings [7,8]. Although DCs are becoming more energy efficient with technological advancement, the expansion of digital services is expected to keep the total energy need of the DC service sector at extremely high levels. With the growth rate of 5.6–6.9 % per year, the DC service sectors’ share in global energy consumption is projected to reach 3–13 % by 2030 [9], and its contribution to global greenhouse gas (GHG) emissions is expected to reach 14 % by 2040 [10]. In 2020 alone, the global DC service sector released approximately 495 M tonnes of GHGs, expressed as CO_2_ equivalents (CO_2eq_) [10]. The annual water consumption per DC reflects the combined need of cooling purposes, heat dissipation during operation (direct use), and electricity production (indirect use), which sums to a serious additional environmental concern [7,11,12].

The environmental sustainability of data centers largely depends on implementing energy-efficient measures, which can be carried out at four distinct levels: site selection level, building design level, equipment level (including IT infrastructure and cooling systems), and data management level (considering resource characteristics) [13]. Recently, Alkrush et al. [14] and Zakarya et al. [15] summarized possible energy efficiency measures to be employed at the latter three levels to reduce the *power usage effectiveness* (PUE) of DCs, with PUE being the main indicator for estimating DC energy consumption, as noted by Ref. [13]. At the site selection level, *the accessibility to free cooling*, and *the opportunities for waste heat recovery* are key for achieving a high energy usage efficiency [16,17]. An additional geographical factor of importance is the *accessibility to renewable energy* [[18], [19], [20], [21]]. However, sustainability of new DCs is impacted not only by energy usage concerns, but also by factors, such as transportation and telecommunication network availability [22], risk factors (e.g., the risk of radioactive contamination of water and earth quakes) [16], financial considerations (e.g., land and construction costs, taxes, political factors (tax incentives, job creation policies), and social factors (e.g., safety and security measures, urban planning regulations) [22]. Due to unique demographic factors, weather conditions, economic preferences, and risk factors specific to different countries, there is no universally accepted approach for evaluating best siting options and sustainability of DCs [13]. Instead, country-specific assessments are commonly utilized. For example, in Portugal, the suitability of data center sites was determined without considering the availability of green power, attributed to its unique electricity distribution system [16].

**Fig. 1:**
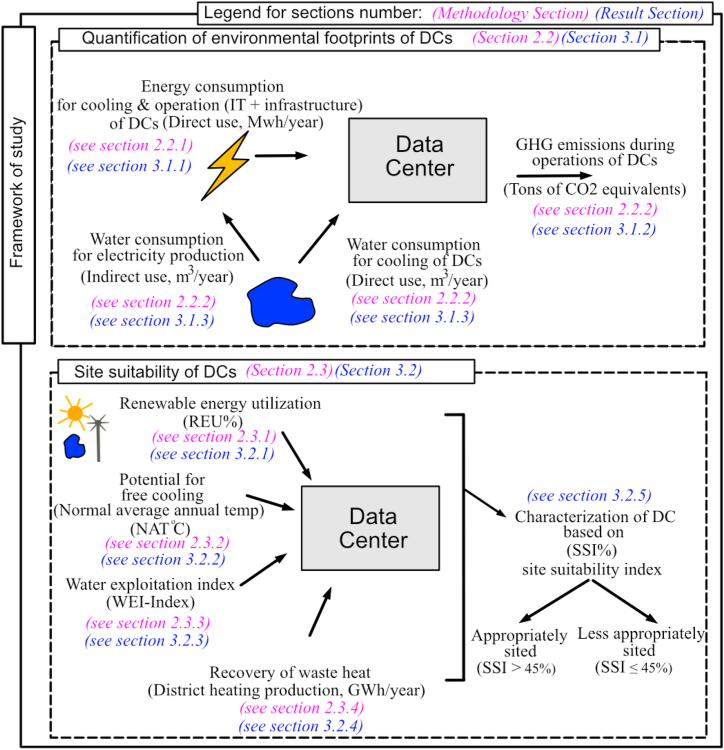
The overall framework of the study.

The current study adopts a geographical focus on Sweden, and exclusively considers geographical factors that represent the environmental attributes of DCs, while financial, risk, political, and social factors are omitted, taking into account that non-environmental attributes of DCs were previously studied by Libertson et al. [23]. This study is unique in that no significant work has been reported in the literature on the environmental site suitability of DCs in Sweden to date. There is an urgent need to investigate environmental attributes of the Swedish DC service sector as several tech companies, like Facebook [24], Amazon Web Services, Microsoft [23], Equinix, DigiPlex, Atnorth [25], Hydro-66 [26], and Hive blockchain [27], are setting up their DCs in Sweden. Currently, little is known regarding the extent to which the Swedish DC service sector is impacting the environment, how well the existing DCs are situated in view of environmental concerns, and which regions should be prioritized for future DC installations. The aim of this study is twofold; firstly, we ***quantify the environmental impact of existing DCs in Sweden*** based on their energy and water consumption and greenhouse gas emissions; secondly, we ***perform a site suitability analysis of the Swedish DCs*** based on their accessibility to renewable energy, possibility of free cooling, resilience to water shortage, and possibility to reuse waste heat. Given the scarcity of pre-existing data on the environmental impact of data centers in the Nordic region in general and in Sweden in particular, adopting this dual approach, i.e., quantification of environmental impact in conjunction with the site suitability analysis, provides a thorough understanding of the spatial variability in environmental impacts of the DC service sector at national level. The methodology adopted and the parameters selected for the analysis in this study are inspired by the research of Shehabi et al. [7], Shehabi et al. [28], and Kheybari et al. [29], but is mainly based on the work of Siddik et al. [12] and Covas et al. [16]. In comparison to these previous studies, however, the methodological approach was extended by the incorporation of two additional parameters, namely the accessibility to renewable energy and the utilization of waste heat, which are important from an environmental perspective [[17], [18], [19], [20], [21]]. These two additional factors compensate the narrow environmental scope adopted by Covas et al. [16] and Siddik et al. [12]. The overall framework and all the parameters used for the analysis in this article are presented in Fig. 1.

To help navigate the reader through the article and to distinctly present the two key segments of the article (i.e., quantification of environmental impact and site suitability analysis of DCs), two key sub-sections are introduced under both the methodology section (see section 2.2, 2.3) and results and discussion sections (see section 3.1, 3.2), and additional third-level sub-headings are employed, corresponding to each performance indicator that was studied, as shown in the framework of the study (see Fig. 1). The insights gained from this study and potential future research are discussed in section 3.3 and conclusions are drawn in section 3.4.

## 2 Materials and methods

**Fig. 2:**
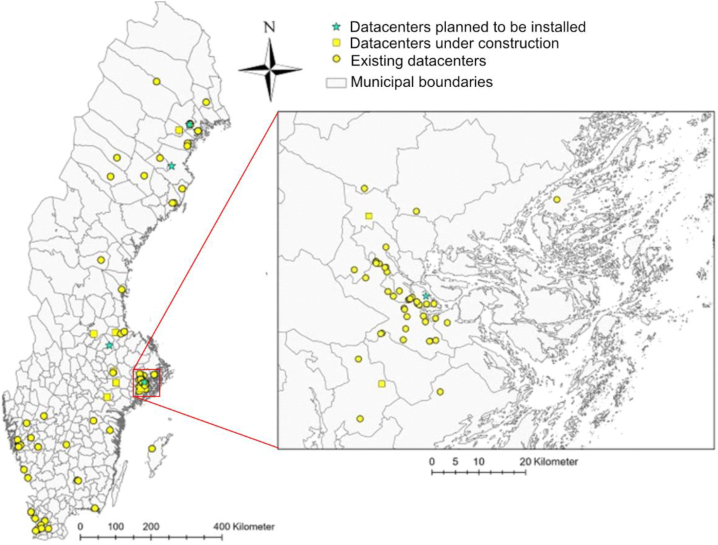
Map of Sweden's municipalities and the Stockholm area (enlarged view), which shows DCs' locations. The map shows 113 unique locations corresponding to 148 DCs operated by 68 companies. These encompass 130 DCs that were in operation in 2022, 14 DCs that were under construction, and 4 DCs that were in the planning phase (GSD Overview map, vector © Lantmäteriet). For 6 DCs, only the name of the municipality was known, and these are represented at the center of the respective municipality.

The analyses of this study are based on 2022 data from 148 DCs in Sweden; 130 currently existing, 14 under construction, and 4 in the planning phase (Fig. 2), which encompass all current and soon-to-be DCs with an installed power of ≥0.3 MW.

### 2.1 Data

The geographical and operational details of DCs, such as location, type of data center (e.g., hyperscale, colocation, cloud, and cryptocurrency mining facilities), their energy usage, water consumption, installed power, footprint area (i.e., the area of the whole data center as well as of server halls), and power usage efficiency (PUE, which Whitehead et al. (2014) [30] define as the ratio of power requirements of the entire data center to the power requirements of the IT equipment), were collected through different means. The sources surveyed in the data collection included: questionnaire surveys (20 DCs), companies' websites (20 DCs), permit applications submitted by companies (8 DCs), web-based data repositories, e.g., datacentermap.com, business-sweden.se, baxtel.se and google.com (37 DCs), and Google Maps (8 DCs). As the DC data was retrieved from several sources, the quality of data for certain parameters varies, e.g., GPS coordinates are accurate for 87 DCs, ambivalent for 20 DCs (approximative coordinates were identified in Google Map by locating street addresses on the companies’ websites) and unknown for 6 DCs (where only the municipality was known).

The data collection for evaluating the site suitability of DCs predominantly involved datasets from governmental sources, namely Statistics Sweden (SCB) and the Swedish Meteorological and Hydrological Institute (SMHI). For details on renewable energy production facilities, including their coordinates, energy output, and installed power capacity, we supplemented our research with data from individual private websites. The validity of these production facilities was corroborated using satellite imagery. A more detailed explanation of the data collection process is provided in each respective section of this article.

ArcGIS Pro was employed for the development of maps and to perform geographical analysis. The digital maps of Swedish municipalities and counties were obtained from the Swedish Lantmäteriets GSD Overview Map [31]. A point vector data layer of the DCs was created in ArcGIS, together with a database that was used to calculate the environmental footprints and site suitability of the DCs using the formulae and criteria presented in section 2.1, 2.2. Various ArcGIS Pro tools such as “Summarize within” and “Spatial join” were used to analyze, summarize, and visualize data of DCs at county level, municipal level, and national level.

### 2.2 Quantification of environmental impact of existing DCs

For quantifying the environmental impact of existing DCs during normal operation, three factors were calculated for each DC: (1) ***energy consumption*** (MWh/year); (2) ***water consumption***, directly for cooling purposes and indirectly for electricity production (**m**^**3**^**/year)**; and (3) ***release of GHGs*** (tonnes of CO_2_eq/year).

#### 2.2.1 Energy consumption of DCs

The methodology applied to assess the annual energy consumption of DCs (*DC_E*_*total*_, MWh/year) depended on the type and form of available data. Despite inconsistencies in the format of available raw data, the aim was to compile a dataset as comprehensive as possible, as deemed important for the work's relevance. To ensure high-quality data, the **first approach** was to use data from primary sources, e.g., from questionnaires or company websites, to obtain values of installed power^1^ of the DCs (IT equipment + other infrastructure, e.g., cooling systems) or installed IT power and power usage effectiveness (PUE). The ***second approach*** was to retrieve information on the installed power from open sources, e.g., media reports and web resources (e.g., datacentermap.com, business-sweden.se, baxtel.se). The **third approach** for estimating DC_Etotal was based on the approach of Strömshed and Perlström. [32], in which data of installed thermal power was obtained from the permit applications for backup power plants of DCs (see Eq. (1)).

(1) $${DC\_E}_{total}=P_{Thermal}*\eta *UR\;*\;\frac{{DC}_{existing}}{{DC}_{planned}}*8760$$

In Eq. (1), $P_{Thermal}$ is the installed thermal power (MW), ${DC}_{existing}$ is the existing number of server halls on the site, and ${DC}_{planned}$ is the number of server halls that, according to the permit application, are to be built on the site. The thermal efficiency η was considered to be 35 % and the utilization rate^2^ ***UR*** was set to 0.765. Multiplication with 8760 converts hourly energy consumption to annual consumption.

A **fourth approach** was adopted for DCs for which information regarding power used by IT equipment was not available, but the DC area was known. In those cases, the value of *DC_E*_*total*_ was calculated using Eq. (2) [12]:

(2) $${DC\_E}_{total}=\left({IT}_{d}/1000\right)\times PUE\times A\times 8760$$

where ***A*** is the area of the DC (in m^2^), either obtained from questionnaire surveys, open-source websites, or estimated using satellite images in Google Maps, and ${IT}_{d}$ is the average power density of the IT equipment (kW/m^2^). For various types of DCs, average values of ${IT}_{d}$ and ***PUE*** were obtained from Shehabi et al. [28] and Shehabi et al. [7], respectively (Table 1). Division with 1000 in Eq. (2) is to convert from kW to MW.

**Table 1:** The average values of **PUE** (power usage effectiveness) and **IT**_**d**_ (power density of IT equipment) for DCs of various sizes, base year 2020. Source: Shehabi et al. [28]; Shehabi et al. [7].

DC area (m^2^)	PUE	IT_d_ (kW/m^2^)
<9.3	2.00	
9.3–92.82	2.35	0.43
46.5–185.7	1.88	0.65
185.8–1858	1.79	0.86
>1858	1.60	1.1
Hyperscale (large effective facilities)^3^	1.13	–

3 Hyperscale facilities are not defined by their size, but rather their manner of operation; they are often among the largest. Therefore, **IT**_**d**_ for high-end facilities is used for hyperscale [10].

#### 2.2.2 Water consumption and GHG emissions

The direct water consumption (${DC}_{direct\;H2O}$, i.e., water consumed directly for cooling purposes) of a certain DC was calculated using Eq. (3) [5], where the energy consumption from section 2.1.1. is multiplied with the DCs’ water usage efficiency, WUE (m^3^/MWh). In Eq. (3), the WUE was set to 1.8 m^3^/MWh, which is the average value of WUEs for all DCs (excluding small server rooms) in the US, according to Shehabi et al. [7].

(3) $${DC}_{direct\;H2O}={DC\_E}_{total}\times WUE$$

The GHG emissions (tonnes CO_2_eq/year), along with the indirect water consumption (m^3^/year) are consequential outputs of the electricity generation that powers DCs. These metrics are determined by considering the aggregate electricity consumption alongside the mean values of GHG emissions and water usage per MWh of electricity produced. Given that electrical energy is usually transported to the nearest outlet point, it is reasonable to access the “mix of electricity consumed by any DC” based on “the electricity production mix” within a defined area, as long as the production covers demand [33]. Thus, average GHG emissions and water consumption per used MWh varies upon the percentage share of different primary energy sources, i.e., hydro, wind, nuclear, and thermal power, in the geographical area where the DC is located. In this study, we applied the approach of Siddik et al. [33] to the four electricity zones that exist in Sweden (see Fig. 5d). For the sake of simplicity of analysis, imports and exports between electricity zones or between neighboring countries were not considered.

The annual *GHG emissions* (*DC*_*GHG*_) and indirect water consumption (${DC}_{indirect\;H2O}$) were determined using Eqs. (4), (5)), following the principles outlined by Siddik et al. [12]. Here, the annual energy consumption (*DC_E*_*total*_) from section 2.1.1. is multiplied by the average GHG emissions per produced MWh (*Avg*_*GHG*_) and water consumption per produced MWh (*Avg*_*H2O*_) within the electricity zone to which the DC is connected. First, however, the variables *Avg*_*GHG*_ and *Avg*_*H2O*_ were calculated using Eqs. (6), (7)), through which the GHG emissions and water consumptions are adapted to the electricity zone within which the data center of interest is located, following the approach suggested by Siddik et al. [33]. This is achieved by multiplying each energy type's contribution to the total energy production (${EP}_{p}/{EP}_{t}$ as per Table 2) by the primary energy source's GHG emissions and water consumption per produced MWh ($F_{p\_GHG}$ and $F_{p_{H2O}}$, as detailed in Table 3), based on life cycle assessment (LCA) data.

(4) $${DC}_{GHG}=DC\_E_{total}\times {Avg}_{GHG}$$

(5) $${DC}_{indirect\;H2O}=DC\_E_{total}\times {Avg}_{H20}$$

(6) $${Avg}_{H20}=\sum \left(F_{p\_H20}\times \left({EP}_{p}/{EP}_{t}\right)\right)$$

(7) $${Avg}_{GHG}=\sum \left(F_{p\_GHG}\times \left({EP}_{p}/{EP}_{t}\right)\right)$$

**Table 2:** The ratio of primary energy sources to the total electricity production for each electricity zone, (column 2 to column 6 obtained from Stymne [34]), and average GHG (**Avg**_**GHG**_) and average water consumption (**Avg**_**H2O**_) per MWh of electricity produced (calculated by using Eq. (6) and Eq. (7), to resolve Eq. (4) and Eq. (5)).

Electricity zone	The ratio of each primary energy source to the total electricity production (${EP}_{p}/{EP}_{t}$)					*Average GHG emissions Avg*_*GHG*_ (kgCO_2_eq/MWh)	*Average H*_*2*_*O consumption Avg*_*H2O*_ (m^3^/MWh)
	Hydro (%)	Wind (%)	Solar (%)	Nuclear (%)	Thermal (%)		
SE1	77.5	16.7	0.03	0	5.8	17.6	10.4
SE2	75.9	19.2	0.1	0	4.8	16.7	10.2
SE3	14.5	11.2	0.9	64.1	9.4	18.4	2.2
SE4	15.1	53.3	3.1	0	28.5	44.4	5.1

**Table 3:** Release of GHG (**F**_**p_GHG**_) and consumption of H_2_O (**F**_**p**_**_**_**H20**_**)** per MWh for various primary energy sources, utilizing LCA data [[35], [36], [37], [38], [39], [40]].

Primary energy source	*F*_*p_GHG*_ (kgCO_2_eq/MWh)	*F*_*p*_*_*_*H20*_ (m^3^/MWh)
Nuclear	6	0.22 (only fuel supply)
Hydro	10.5	13.3
Wind	15.5	0.005
Solar (PV)	27	0.38
**Thermal power (**x **all below)**	**118**	1.75 (excluding fuel supply)
Biofuel	23	
Waste	222	
Fossil fuels	601	

The *F*_*p_GHG*_ values were retrieved from Gode et al. [35] and Vattenfall [[36], [37], [38], [39]]. The contribution from thermal electricity production was estimated based on the proportion of different fuels employed in 2020, specifically biofuels (70.8 %), municipal waste (19.4 %), and fossil fuels (9.8 %) [34]. This estimation resulted in an average *F*_*p_GHG*_ of 118 kgCO_2_eq/MWh (see Table 3). The GHG emissions for the fossil component was estimated based on the average *F*_*p*_*_*_*GHG*_ for natural gas, coal, and oil from Gode et al. [35], corresponding to 601 kgCO_2_eq/MWh. The *F*_*p_H2O*_ values were obtained from Mekonnen et al. [40]. Since *F*_*p_H2O*_ from hydropower plants is strongly influenced by climatological factors, it is reasonable to calculate the average value of *F*_*p_H2O*_ by taking the ratio of the total water consumption by hydropower and the total electricity production from hydropower in northern Europe [40]. Only the fuel supply phase was included for nuclear power, since seawater is primarily used in the operations of nuclear reactors in Sweden [41]. For all types of thermo-electric power, *F*_*p_H2O*_ values for only the operational phase were considered (i.e., only direct water use while burning different fuels to produce electricity).

### 2.3 Site suitability of DCs

Placing DCs in favorable environmental conditions effectively streamlines infrastructure processes and reduces environmental impact [12,16,29]. For example, strategically siting new DCs in the USA was estimated to reduce the water scarcity footprint and GHG emissions by up to 90 % and 55 %, respectively [12]. Considering this, the site suitability analysis of DCs in this study was performed using four factors that were assessed at municipality level: (a) **F1-Percentage renewable energy utilization** (REU%, i.e., The ratio of total electricity consumption to the production from wind, hydro, or biomass sources in the county where the municipality is situated); (b) **F2-**Support **of climatic conditions to implement free cooling in the municipality,** here assessed in terms of the normal average annual temperature (NAT, ^o^C); (c) **F3-Resilience to water shortage,** here assessed in terms of the regional (county level) water exploitation index (WEI, %); and (d) **F4-Potential for recovery of waste heat,** which was characterized by the district heating production in the municipality (DHP, GWh/year). Thus, factors F2 and F4 were determined for each individual municipality directly, while factors F1 and F3 were initially calculated at county level whereafter all the municipalities in a certain county were assigned the same scores. Each of the four factors was assigned a score ranging from 1 to 10 for every municipality in Sweden, following the principles specified in Table 4. Thus, while considering these four factors (F1–F4) together, any municipality can score a minimum of 4 and a maximum of 40. The ratio of the sum of these 4 scores to the maximum attainable score (i.e., 40) is defined as the site suitability index (SSI) for a certain municipality. A higher score indicates greater suitability for DC installation. Thus, the SSI values can help distinguish environmentally favorable DC locations from less suitable ones.

**Table 4:** Criteria for assigning scores to municipalities in the site suitability analysis, with respect to normal average annual temperature (**NAT**), renewable energy utilization (**REU**), water exploitation index (**WEI**), and district heating production (**DHP**).

REU (%)	Score	NAT (°C)	Score	WEI (%)	Score	DHP (GWh/year)	Score
942–1046	1	8.2–9.2	1	4.1–4.5	1	0–498	1
839–942	2	7.2–8.2	2	3.6–4.1	2	498–996	2
736–839	3	6.2–7.2	3	3.2–3.6	3	996–1495	3
632–736	4	5.3–6.2	4	2.8–3.2	4	1495–1993	4
529–632	5	4.3–5.2	5	2.3–2.8	5	1993–2491	5
425–529	6	3.3–4.3	6	1.9–2.3	6	2491–2989	6
322–425	7	2.3–3.3	7	1.4–1.9	7	2989–3487	7
219–322	8	1.3–2.3	8	1.0–1.4	8	3487–3986	8
115–219	9	0.3–1.3	9	0.5–1.0	9	3986–4484	9
12–115	10	−0.7–0.3	10	0.1–0.5	10	4484–4982	10

#### 2.3.1 Accessibility to renewable energy

The enhanced use of renewable energy is a cost-effective option [42]. In this study, the degree of accessibility of DCs to renewable energy is characterized by the percentage renewable energy utilization (***REU***), defined as the ratio of total electricity consumed to the total renewable energy produced in a county (Eq. (8)). The lower the value of REU, the higher the availability of renewable energy in that county.

(8) $$REU=\frac{Electricity\;use\;\left(TWh/år\right)}{Produced\;RE\;\left(TWh/år\right)}\;*\;100$$

The percentage REU was calculated at the county level because it represents the highest geographical resolution available for estimating the electricity mix, specifically the proportion of electricity generated from renewable sources. Conducting the analysis at county level rather than at the national level made it possible to account for transmission losses and limitations in Sweden's main and regional networks, which are generally characterized by power deficits in the south and surpluses in the north [23,[43], [44], [45], [46]]. Considering these limitation in the capacity of the electric grid, the accessibility to renewable energy is crucial both for minimizing CO_2_ emissions from electricity production and for mitigating the risk of power shortages that otherwise would necessitate the reliance on fossil-based backup power plants. In the analysis, we assume that the electricity usage mix in a given county mirrors the electricity production mix in the same county, provided that production adequately meets the local demand [33].

**Fig. 3:**
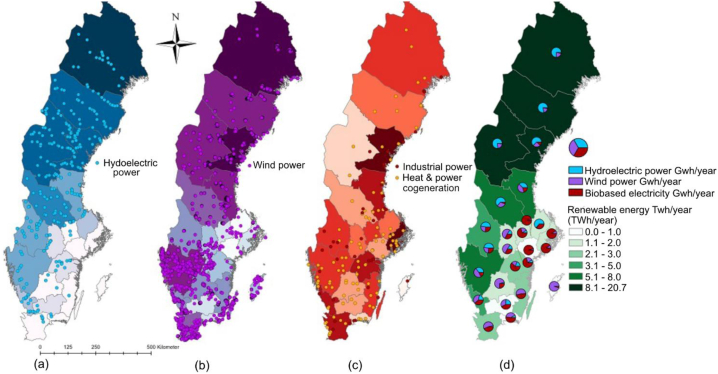
a–d. The geographical location of electricity-producing facilities: (a) hydro, (b) wind, (c) biopower, and (d) the total renewable energy production and the share of each type of energy (TWh/year) (GSD Overview map, vector © Lantmäteriet).

The data on electricity usage in Swedish counties was obtained from SCB [47]. To estimate the total renewable energy production in a specific county, we compiled a database in ArcGIS. This database encompassed all renewable energy production facilities in Sweden, with information on their geographical coordinates (WGS84) and annual renewable energy output (GWh/year). It encompassed 345 hydropower plants (with an output power exceeding 2 MW, from vattenkraft.info [48]), 4600 wind turbines (installed across Sweden before 2022, as reported by Vindbrukskollen [49]), 152 biopower plants, and 42 other biopower-producing industries (according to Bioenergitidningen [50]). Using this dataset, the average production of renewable electricity in each county was calculated in ArcGIS using the summarize tool. As expected, the results show that the production of renewable energy is higher in the northern parts compared to the southern parts of the country (see Fig. 3a–d). Furthermore, it is deduced that hydroelectric power is prominent in northern Sweden, wind power dominates in the southwest, and the majority of biobased electricity being produced in the southeast of Sweden.

#### 2.3.2 Potential for free cooling

Establishing DCs in regions with colder climates can significantly decrease the reliance on energy-extensive cooling machines. This results in a substantial reduction in both electricity and water consumption for cooling processes [12]. Thus, the ambient outdoor temperature is known to strongly influence the power use efficiency (PUE) of DCs [51]. We assessed the suitability of each municipality for implementing free cooling in data centers through the normal average ambient temperature (NAT). A lower NAT is desirable as it means that free cooling can be used for a longer period of the year [51]. The NAT data was obtained from the SMHI's compilation of annual average ambient temperatures for the normal period 1991–2020 from 708 weather stations [52]. For municipalities that have multiple weather stations, an average NAT value was calculated. For municipalities that lack NAT data, the data of the nearest weather station located outside the municipal boundary was considered.

An outdoor temperature below 20.2 °C has the capability to cool IT equipment using indirect airborne free cooling [53]. Therefore, to further illustrate the possibility of free cooling, the average number of hours per year with an ambient temperature below 20° was calculated for municipalities for which SMHI compiled historical hourly temperature data for the period 2011–2020 [54].

#### 2.3.3 Resilience to water shortages

Sweden has abundant fresh water resources [55]; nevertheless, water shortages occur periodically, especially in the coastal areas in the southeastern region of the country [56]. Considering that DCs require water, it is important to illustrate the capacity of a geographical area to meet water needs for planned and existing DCs. Two indices, the water exploitation index (WEI) and the water exploitation index plus (WIE+), are reported in the literature for estimating water utilization rates in specific areas [56]. The WEI is calculated as the ratio of the average water usage to the average available renewable freshwater resources within a specific geographical area (Eq. (9)), while the WEI + also accounts for returns from water usage, thus better reflecting the net water abstraction. According to the European Environment Agency (EEA), water resources in a region are considered under stress when the WEI + falls between 20 % and 40 %. An WEI + exceeding 40 % indicates unsustainable water resource usage [57]. However, since data regarding the amount of water returned to water reservoirs is usually not available, most site suitability analyses rely on the WEI index [56].

(9) $$WEI=\frac{Withdrawn\;water}{Available\;water}$$

Data of available water, stated per county, was obtained from Appendix 10 of SMHI's report Hydrology no. 126 (base years 1981–2010) [58]. This data represents annual average values, even though there is logically a substantial temporal variability, and areas that typically have low WEIs can experience periodic water shortages [56]. The available water is also a difficult parameter to quantify. Each county includes several catchment areas with different conditions, and the available water depends on the local runoff together with inflows and outflows from surrounding regions and outflow to the sea [58].

Data on water withdrawal per county (base year 2015) was obtained from Moström [56]. Water used by hydropower facilities was excluded from the dataset, as it is considered in-situ use with a low proportion of net water withdrawal [58]. The statistics describing water usage mainly consider freshwater, as saltwater does not affect drinking water status. For this reason, nuclear power is also excluded from the water withdrawal statistics [59].

#### 2.3.4 Potential for recovery of waste heat

Most of the electrical energy used by IT equipment and support processes is ultimately emitted as excess heat [60]. Significant energy savings at societal level are possible if this waste heat is recovered, for example via the district heating (DH) network [61]. Many municipalities in Sweden possess well-developed DH networks, which contribute to approximately 60 % of the energy utilized for heating and hot water generation in residential buildings and facilities in Sweden. Consequently, distributing excess heat from DCs to these DH networks represents a logical approach [47,62]. Several DCs in Stockholm, owned by, e.g., Ericsson, Bahnhof, Glesys, and Interxion, sell waste heat to Stockholm Exergi, which aims to utilize DCs' excess heat to serve 10 % of the city's total DH demand [63]. The distance between a DC and the nearest DH network connection point is, however, critical, and a maximum distance of 1000 m has been suggested to keep connection costs at a reasonable level. Additionally, the DH network's capacity to utilize the amount of heat released by the connected DC is essential (personal communication with Börje, 2021).

Since geographical coordinates of DH plants and digital maps of DH networks are difficult to extract, the potential for connecting a DC to a DH network in a certain municipality was estimated from data of district heating production (DHP, GWh/year) in that municipality. The rationale here is that a higher DHP should indicate a more extensive district heating network, offering more potential connection points between the DCs and the DH network within that municipality. An annual average DHP for each municipality was calculated using data from SCB on fuel input for DHP for the years 2010–2019 [47]. To keep the analysis simple, it was assumed that neighboring municipalities do not share DH networks.

## 3 Results and discussion

**Fig. 4:**
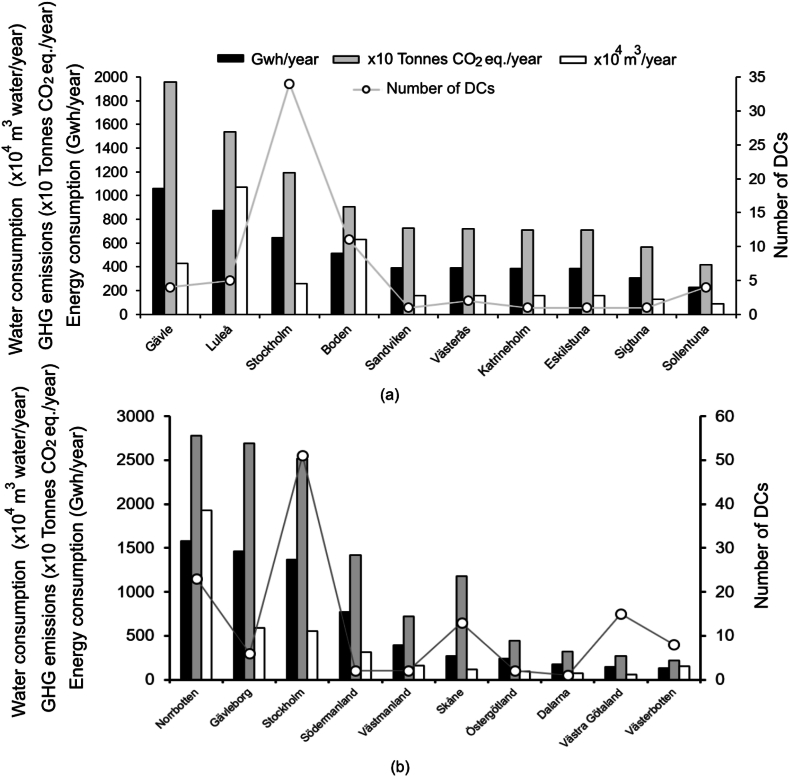
Electricity consumption (GWh/year), GHG emissions (tens of tonnes CO_2_eq/year), water consumption (10,000 m^3^/year) (primary axis), and number of DCs per county (secondary axis) (a) for the 10 municipalities and (b) for the 10 counties where DCs have highest electricity usage.

**Fig. 5:**
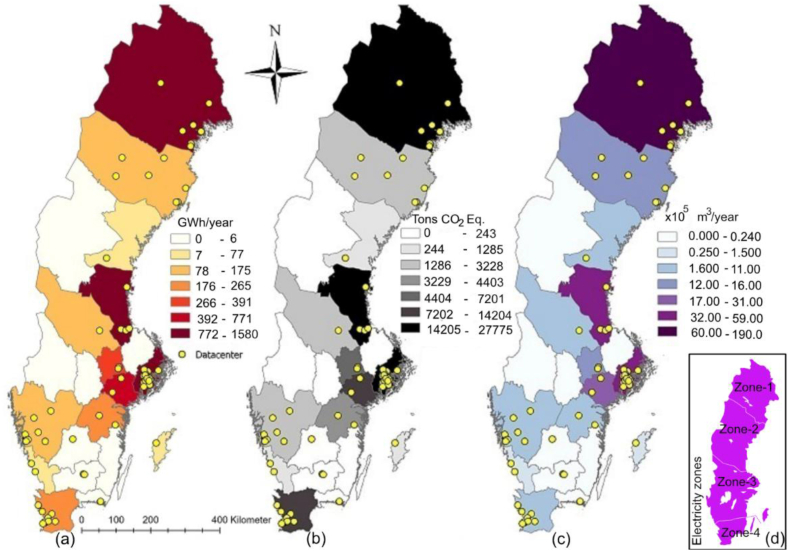
a–c. Location of existing DCs, with geographical variation in electricity usage (GWh/year), release of GHG emissions (tonnes CO2eq/year), and water consumption (m^3^/year) by DC service sector in various counties in Sweden. (Fig. 5d in the inset shows the 4 electricity zones in Sweden, GSD Overview map, vector © Lantmäteriet.).

**Fig. 6:**
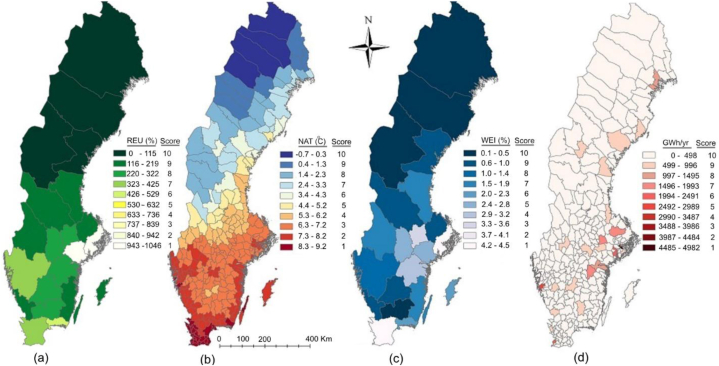
a–d. From left to right. Sweden's counties color graded on the basis of renewable energy utilization (REU), normal average annual temperature (NAT), water exploitation index (WEI), and district heating production (DHP) (GSD Overview map, vector © Lantmäteriet). (For interpretation of the references to color in this figure legend, the reader is referred to the Web version of this article.)

**Fig. 7:**
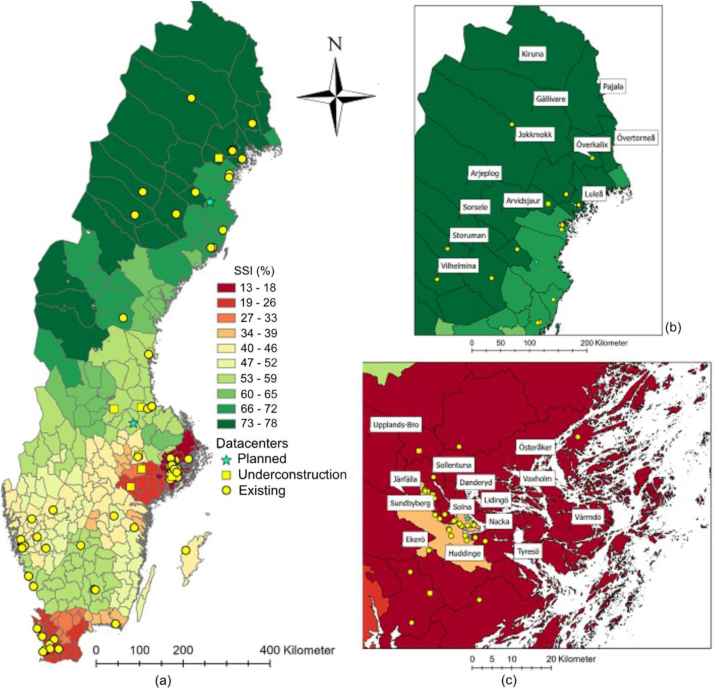
a–c. Location of existing, planned and DCs under construction, together with geographical variation of site suitability index (SSI) among Sweden's municipalities (enlarged view of Northern part (Fig. 7b) and Stockholm region (Fig. 7c)) (GSD Overview map, vector © Lantmäteriet).

The first part of the results and discussion section details the environmental impact of existing DCs (section 3.1) by examining electricity usage of DCs (see Fig. 4, Fig. 5a), annual GHG emissions (see Fig. 5b) and total water consumption (Fig. 5c). The second section (section 3.2) identifies optimal locations for future DCs through a multi-criteria site suitability analysis of Swedish municipalities (see Fig. 7), guided by four performance indicators (F1–F4): (F1) renewable energy utilization, measured by the ratio of total electricity consumed to total renewable energy produced (Fig. 6a); (F2) potential for free cooling, based on normal annual ambient temperatures (Fig. 6b); (F3) resilience to water shortages, assessed via the WEI (Fig. 6c); and (F4) potential for waste heat recovery, quantified as total district heating production (Fig. 6d). Reflections on the methodology used and limitations of the study are provided in section 3.3.

### 3.1 Quantification of environmental impact of existing DCs

#### 3.1.1 Energy consumption by DC service sector

Among the 130 operational Swedish DCs analyzed for this study, the annual electricity consumption (*DC_E*_*total*_) varied between 1.4 and 876 GWh/year, with an average of 84 GWh/year. Taken together, the DCs in Sweden have a total power output of approximately 760 MW, which corresponds to an electricity usage of around 6.7 TWh/year. The total electricity consumption of the entire Swedish DC service sector amounted to 5.4 % of the total national electricity usage in 2020 (125 TWh [34]) and is equivalent to 3.3 % of the global DC sector's electricity consumption (205 TWh [64]). Fig. 4, Fig. 5a illustrate the electricity consumption associated with DC facilities in the different counties of Sweden during 2020. The highest value is found in Norrbotten (1580 GWh/year), followed by Gävleborg (1460 GWh/year) and Stockholm (1370 GWh/year) (Fig. 4). The combined electricity consumption of the DC sector in these three counties accounts for approximately 66 % of the sector's total electricity usage on national level. Approximately 78 % of the sector's electricity consumption (∼6500 GWh) is related to the 10 municipalities presented in Fig. 4a, where server halls of various IT giants, e.g., Microsoft, AWS, and Facebook, are located. This indicates that the growth of the DC service sector is not uniform throughout Sweden. Furthermore, identifying the municipalities where DC service sector predominantly exists helps in pinpointing the regions primarily facing the environmental consequences posed by the Swedish DC service sector.

#### 3.1.2 GHG emissions by DC service sector

The GHG emissions resulting from the Swedish DC's electricity consumption were assessed to vary from 52 to 15,404 tonnes CO_2_eq/year when individual DCs are considered. The average DC accounted for 1632 tonnes CO_2_eq/year. The total annual GHG emissions of the entire sector amounts to approximately 1.3 × 10^5^ tonnes of CO_2_eq/year, equaling 0.3 % of Sweden's total GHG emissions in 2020 (46.3 × 10^6^ tonnes CO_2_eq [65]) and 0.03 % of the global DC sector's GHG emissions (495 × 10^6^ tonnes CO_2_eq [10]). Therefore, while the DC service sector in Sweden contributes significantly to global DC energy usage (3.3 %; see section 3.1.1), its contribution to global GHG emissions is a hundredfold lower. This suggests that Sweden is a highly favorable location for the establishment of DCs on a global scale. The percentage share of the Swedish DC service sector in the national GHG emission inventory (0.3 %) is of a similar order of magnitude as that presented for the US (0.5 %) [12]. Fig. 5b shows the distribution of GHG emissions related to DCs for different counties. Highest GHG emissions was found for Norrbotten (2.8 × 10^4^ tonnes CO_2_eq/year), followed by Gävleborg (2.7 × 10^4^ tonnes CO_2_eq/year) and Stockholm (2.5 × 10^4^ tonnes CO_2_eq/year) (Fig. 4b), and the vast majority of the GHG emissions, about 73 % (∼9.5 × 10^4^ tonnes CO_2_eq/year), can be related to only 10 municipalities. Generally, the geographical distribution of high and low GHG emission regions aligns with regions of high and low electricity usage (Fig. 4).

Northern parts of Sweden (the region comprised of electricity zones SE1 and SE2) are more preferrable for siting of DCs, as the electricity here is mainly generated from hydropower and to some extent from wind power, thus causing low GHG emissions. Nuclear power has a major share in zone SE3, which also results in low emissions, much like SE1 and SE2, though has comparatively fewer possibilities of free cooling. Conventional thermal power has a larger share of the electricity production in zone SE4, in the southernmost part of the country, which therefore has the highest GHG emissions per MWh of electricity produced (44.4 kgCO_2_eq/MWh). This is demonstrated in Fig. 4b through the bars showing energy use and GHG emissions from DCs in Skåne, which is thus the least preferable zone for siting of DCs both in relation to GHG emissions and possibility of free cooling. Fig. 5 illustrates that DCs in Skåne County consume significantly less energy compared to DCs in Norrbotten County, yet their GHG emissions are relatively similar.

#### 3.1.3 Water consumption by DC service sector

The water consumption of Swedish DCs varies between 6.1 × 10^3^ and 1.1 × 10^7^ m^3^/year. The total water consumption of the sector was estimated to about 4.2 × 10^7^ m^3^/year, which is approximately 14 % of the water consumed by the whole Swedish industrial sector (3 × 10^8^ m^3^; excluding hydropower) (base year 2020 [41]), and 12 times less than that of US DC service sector (5.13 × 10^8^ m^3^ [16]). This contrast is mainly due to the fact that the electricity consumption of the DC service sector in the US (73 TWh in 2020 [11]) is 11 times higher than that of Sweden (6.7 TWh). Likewise electricity utilization and GHG emissions, water consumption figures are highest for Norrbotten (1.9 × 10^7^ m^3^/year; see Fig. 5c), accounting for 45 % of the total water consumption of the DC sector in Sweden. The geographical location of the DCs significantly impacts their water footprints, with energy production in regions SE1 and SE2 consuming approximately 4 and 5 times more water per produced MWh, respectively, compared to SE3 and SE4. This difference is due to the large hydropower contribution to the electricity mix in northern Sweden (see Fig. 4).

### 3.2 Site suitability of DCs

#### 3.2.1 Renewable energy utilization (REU)

Fig. 6a shows the geographical variation in REU, presented at county level. Ideally, DCs should be located in areas that have a REU below 100 % (i.e., consumption of electricity < production of renewable energy), i.e., with a score of 9–10 according to Table 4. This is the case in Jämtland (12 %), Västerbotten (27 %), Norrbotten (40 %) and Västernorrland (52 %). The utilization of renewable energy was found to be highest in the Stockholm County (>1000 %), which means that the demand is more than 10 times the amount of RE produced. Fig. 6a shows that Northern Sweden has a high production of renewable energy and a low energy consumption, while the trend for Southern Sweden is the opposite.

#### 3.2.2 Normal average annual temperature (NAT)

As shown by the NAT data presented for all Swedish municipalities in Fig. 6b, there are three municipalities that receive a score of 10 with regard to temperature. These are all located in the northernmost county (Norrbotten) and include Kiruna (−0.7 °C), Arjeplog (−0.1 °C), and Gällivare (0 °C). The highest NATs, corresponding to a score of 1, were found in the municipalities of Vellinge (9.2 °C) and Malmö (9.1 °C), which are located in the county of Skåne in the southernmost area of the country. The number of hours per year below 20 °C is highest in Kiruna (8611 h/year), and lowest in Malmö (8124 h/year). It is concluded that free cooling can cover the entire cooling demand from a minimum of 93 % (8124 h/year, in southernmost Malmö) to a maximum of 98 % (8611 h/year, in northernmost Kiruna) of the year (8760 h), depending on the location in Sweden. These results can be compared with the findings of Covas et al. [16], who report an average of 7300 h/year below 21 °C in Portugal, indicating that Sweden has at least 1300 h more of free cooling than available in Portugal.

#### 3.2.3 Water exploitation index (WEI)

The share of the available water resources that are withdrawn (excluding hydropower), or the WEI values for the counties in Sweden, vary between 0.06 % and 4.5 % (Fig. 6c). This is significantly less than the average WEI+ of all European nations, which falls between 15 % and 25 % [57]. The most optimal WEI values in Sweden are observed in the northern counties, specifically Jämtland (0.06 %), Västerbotten (0.2 %), and Norrbotten (0.4 %), all of which received a score of 10 (Table 4). The highest proportions of water resource withdrawals are evident in the counties of Stockholm (4.5 %) and Skåne (4.2 %), which are both assigned a score of 1.

#### 3.2.4 District heating production (DHP)

In the analysis of DHP among Swedish municipalities (Fig. 6d), Stockholm stands out with the highest annual average DHP at 4982 GWh/year, which equates to a score of 10. In comparison, Gothenburg, the second-highest producer, has an annual output of 2685 GWh/year, corresponding to a score of 6. Notably, 36 municipalities do not have any DHP, which is reflected in their score of 1, and 268 municipalities have a DHP below 498 GWh/year (Fig. 6d).

To summarize, Fig. 6a–d shows that the renewable energy utilization (REU), normal average annual temperature (NAT), and water exploitation index (WEI) have more geographical variation, thus, more strongly influence the site suitability score, than the district heating production (DHP).

#### 3.2.5 Overall site suitability index (SSI)

The results of the site suitability analyses, after having combined the results from the four individually assessed factors, revealed SSI values for the municipalities in Sweden ranging from 13 % to 78 %, with an average of 45 %. Table 5 presents the results for the 12 municipalities with the highest SSI score. It is concluded that the municipalities of Arjeplog, Gällivare, Jokkmokk, and Kiruna in the Norrbotten county have the best conditions for establishing DCs, with achieved SSIs of 78 %. Fig. 7a–c presents the geographical variation of the SSIs for all municipalities in Sweden.

**Table 5:** The values for normal average annual temperature (NAT), renewable energy utilization (REU), water exploitation index (WEI), and district heating production (DHP) for the 12 municipalities that achieved the highest value for site suitability index (SSI).

Municipality	County	NAT (°C)	WEI (%)	REU (%)	DHP (GWh)	Score	SSI (%)
Arjeplog	Norrbottens län	−0.1	0.4	40	0	31	78
Gällivare	Norrbottens län	0.0	0.4	40	199	31	78
Jokkmokk	Norrbottens län	0.2	0.4	40	47	31	78
Kiruna	Norrbottens län	−0.7	0.4	40	238	31	78
Arvidsjaur	Norrbottens län	0.9	0.4	40	46	30	75
Luleå	Norrbottens län	2.6	0.4	40	1002	30	75
Pajala	Norrbottens län	0.5	0.4	40	28	30	75
Överkalix	Norrbottens län	1.3	0.4	40	34	30	75
Sorsele	Västerbottens län	0.5	0.2	27	20	30	75
Storuman	Västerbottens län	0.7	0.2	27	37	30	75
Vilhelmina	Västerbottens län	0.8	0.2	27	65	30	75
Övertorneå	Norrbottens län	1.1	0.2	40	34	30	75

**Fig. 8:**
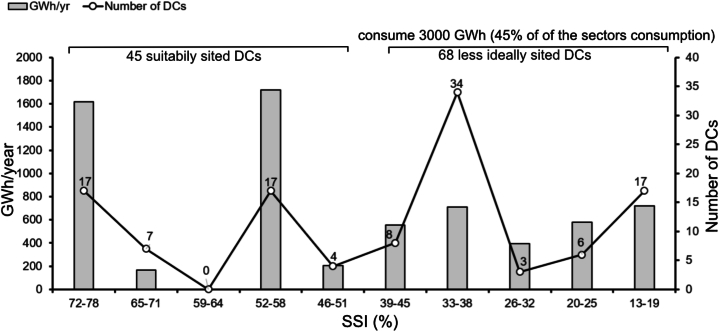
The frequency distribution of DCs and associated electricity consumption (GWh/year) with respect to SSI intervals (%). SSI ≤45 % constitute less ideally sited DCs.

Fig. 7 shows that municipalities in northern Sweden are more suitable for siting of DCs as they have a high share of renewable energy in the energy mix, lower atmospheric temperature (below 20 °C), and sparse population, which means less water and energy usage. On the other hand, the municipalities in the south of Sweden, in the county of Skåne, are less suitable for siting of DCs because their energy mix contains less renewable energy, the atmospheric temperatures are higher, the water resources are limited, and the region is densely populated. All 14 municipalities in the county of Stockholm also have low SSI values, between 13 % and 18 % (Fig. 7c). Fig. 8 demonstrates the extent to which existing DCs are optimally sited and presents the frequency distribution of DCs and their total electricity consumption with respect to various SSI intervals (13%–78 %). The mean value of SSI is 45 %; using this value, DCs in Fig. 8 are split into two main groups, namely suitably sited (45 DCs with 45 ≤ SSI ≤78) and less ideally sited (68 DCs with 13 ≤ SSI ≤45). The latter is a group where less attention was paid to the potential of the geographical site with respect to availability of renewable energy, free cooling, water resources, and reusability of waste heat. Data shows that the energy consumption of the less ideally sited DCs (SSI ≤45 %), corresponds to 3000 GWh, which is 45 % of the whole sector's energy consumption, indicating the long-term negative impact of poorly sited DCs. Fig. 8 shows that there are only 17 DCs that attained highest SSI score of 72–78 and this score could be considered as a touch stone for the site selection of any new DC in the future.

Looking at Fig. 8 together with Fig. 4, Fig. 7 reveals that most (13 of 17) DCs that have an SSI between 72 % and 78 % are located in Norrbotten county. High electricity consumption is found especially in the municipalities of Luleå and Boden, which received favorable index values of 75 % and 73 %, respectively (Fig. 4).

### 3.3 Reflections and limitations

Sweden is recognized as a generally advantageous location for establishing DCs. However, this study highlights significant regional variations within the country, underscoring the increasing importance of conducting site suitability analyses in the context of climate change and the anticipated growth of electricity-intensive industries. The principal findings are centered on Sweden, thereby primarily benefiting stakeholders engaged in decision-making for evaluating existing or strategically planning new DCs within this country, incorporating a comprehensive environmental perspective. Nevertheless, the gathered data on GHG emissions, water consumption, and individual site suitability parameters could be valuable to a diverse array of stakeholders, including field experts, academic institutions, and businesses.

Collecting data on parameters such as GPS coordinates and energy consumption from varied sources introduces inconsistencies attributable to disparate methodologies, definitions, and measurement standards. Such variability complicates data aggregation and comparison, affecting analysis reliability. As the number of data sources grows, the challenge of ensuring data accuracy and reliability increases. Nevertheless, in this context, the precision of GPS coordinates was considered non-critical, given that calculations and analyses were conducted at the municipality or county level. Crucially, acquiring data on electricity usage across a broad spectrum of DCs was essential, particularly since individual large DCs can by themselves contribute significantly to the sector's overall environmental impact. Consequently, leveraging multiple data sources augmented the analysis, offering a thorough perspective on DC operations and environmental implications. This approach additionally revealed patterns and insights that might remain obscured when relying solely on a singular data source.

This study aims to enrich the existing body of literature by providing a detailed environmental perspective of the Swedish DC service sector. For future studies, it is recommended to enhance the environmental factors used in this study with additional factors such as political, economic, social, and risk factors, among others, to perform a more comprehensive site suitability analysis of DCs. Additionally, there is need for more case-study based research to provide updated and accurate template key figures.

### 3.4 Conclusion

In this study, environmental footprints of DCs in Sweden are estimated and a new approach for determining the site suitability of DCs is presented. It is estimated that the Swedish DC service sector annually consumes 6.7 TWh of electricity (≈5.4 % of the national electricity consumption), 4.2 × 10^7^ m^3^ of water (≈14 % of the industrial sector's total water footprint), and releases 1.3 × 10^5^ tonnes of CO_2_eq (0.3 % of the national GHG emissions). In the study, 68 non-ideally sited DCs are identified, characterized by a site suitability index (SSI) ≤ 45 %, where the availability of renewable energy, access to free cooling and to water resources, and potential for waste heat utilization was not optimal. The most favorable conditions for siting of DCs are found in northern Sweden, especially in municipalities located in the counties of Norrbotten, Jämtland, and Västerbotten (73 % ≤ SSI ≤78 %). In view of the rapidly changing climate, strategically siting of new DCs is likely to become increasingly important for reducing the environmental impact of the DC service sector.

## CRediT authorship contribution statement

**Kim Jerléus:** Writing – review & editing, Writing – original draft, Formal analysis, Data curation. **Muhammad Asim Ibrahim:** Writing – review & editing, Writing – original draft, Supervision, Project administration, Methodology, Data curation, Conceptualization. **Anna Augustsson:** Writing – review & editing.

## Declaration of competing interest

The authors declare that they have no known competing financial interests or personal relationships that could have appeared to influence the work reported in this paper.

## Footnotes

1 The actual operable power is often below the installed power as free cooling is available for most of the year.

2 Assuming that the generators are running at 85 % of maximum capacity, with 10 % used as reserve units [(0.85 x 0.9) = 0.765] [32].
