# Supplementary material for: Environmental footprints of the data center service sector in Sweden
Source: Heliyon. 2024 May 18;10(11):e31290. doi: 10.1016/j.heliyon.2024.e31290 (PMC11140606; doi:10.1016/j.heliyon.2024.e31290)
Supplement: Multimedia component 1 [file mmc1.docx]

Appendix A - questionnaire

In the first part of the questionnaire survey, general questions were found for each company, these are illustrated in Table A1 below. Because confidentiality was promised, the names of the companies were not stated, instead each company was assigned a number. In the first question, the respondents were asked to rank how important different location factors were during site selection. This is based on the interval: not at all important (1), less important (2), neutral (3), quite important (4) and very important (5). The numbers in parentheses were used to later calculate the mean for each factor. The following factors were examined:

- Cold climate (KK)
- Cheap electricity (BE)
- Access to renewable energy (TFE)
- The possibility to use excess heat (MTS)
- Protection against natural disasters (SMN)
- Protection against intrusion (SMI)
- Good connection possibilities (GU)
- Proximity to users (NA)

The result is summarized in Figure A1 below:

**Figure A1.** Mean value for each localization factor, based on ranking of 15 respondents from the survey. Error bars show standard deviation.

The second inquiry focused on the recovery of excess heat in data centers, presented as a straightforward yes/no question. Respondents confirming the utilization of waste heat were requested to specify the methods employed. The subsequent third and fourth questions explored the use of free cooling systems and the total number of data centers operated by the company. The remaining questions, compiled in Table A2 and Table A3, concerned the collection of technical data specific for each data center. Technical data were then compiled together in appendix B, together with data taken from other sources, to calculate energy use.

**Tabell A1.** Part 1 of the questionnaire survey. Consisting of general information on localization factors, recovery of waste heat, use of free cooling and the number of data centers.

| **Company** | **1) Location factors** | | | | | | | | **2) Reuse of excess heat** | | **3) Free cooling** | | **4) Number of DC** |
| --- | --- | --- | --- | --- | --- | --- | --- | --- | --- | --- | --- | --- | --- |
|  | **KK** | **BE** | **TFE** | **MTS** | **SMN** | **SMI** | **GU** | **NA** | **2a) Yes/no** | **2b) Method** | **3a) Air** | **3b) Water** | **Number** |
| 55 | 4 | 4 | 4 | 4 | 4 | 4 | 4 | 1 | Yes | Green house, resident heating | Yes | No | 2 |
| 37 | 5 | 5 | 5 | 4 | 4 | 4 | 5 | 2 | Yes | Local heating, wood chip dryer | Yes | No | 2 |
| 7 | 4 | 5 | 5 | 5 | 3 | 3 | 5 | 3 | Yes | District heating | No | No | 1 |
| 31 | 1 | 2 | 5 | 5 | 3 | - | 4 | 2 | Yes | District heating | No | No | 2 |
| 38 | 5 | 5 | 5 | 5 | 5 | 5 | 5 | 3 | No | investigates suitable projects | Yes | No | 1 |
| 51 | 2 | 4 | 3 | 3 | 3 | 5 | 5 | 5 | Yes | Heating office space | Yes | No | 1 |
| 41 | 5 | 4 | 3 | 3 | 5 | 5 | 5 | 2 | No |  | Yes | No | 1 |
| 20 | 2 | 5 | 4 | 4 | 5 | 4 | 5 | 4 | Yes | Local heating | Yes | No | 1 |
| 46 | 1 | 1 | 5 | 5 | 5 | 5 | 5 | 5 | Yes | District heating | Yes | No | 2 |
| - | 3 | 3 | 4 | 3 | 4 | 5 | 5 | 5 | No |  | No | No | 4 |
| 68 | 5 | 5 | 5 | 5 | 5 | 5 | 5 | 5 | No |  | Yes | No | 1 |
| 4 | 1 | 1 | 3 | 1 | 2 | 5 | 5 | 3 | No |  | Yes | No | 1 |
| 35 | 4 | 5 | 4 | 3 | 4 | 5 | 4 | 3 | No |  | Yes | No | 2 |
| - | 3 | 4 | 5 | 4 | 4 | 5 | 5 | 3 | No |  | Yes | No | 1 |
| 17 | 5 | 5 | 5 | 5 | 5 | 5 | 5 | 4 | Yes | District heating | Yes | Yes | 1 |
| Average | 3,3 | 3,9 | 4,3 | 3,9 | 4,1 | 4,6 | 4,8 | 3,3 |  |  |  |  |  |
| SD | 1,6 | 1,5 | 0,8 | 1,2 | 1,0 | 0,6 | 0,4 | 1,3 |  |  |  |  |  |

**Table A2.** Part two of the questionnaire. Specific technical data for each company's data center.

| **Company** | **Type of DC** | **Municipality/adress** | **Ptot (MW)** | **Pit (MW)** | **PUE** | **Total surface area** | **Server area** | **H_2_O m^3^/år** | **WUE** |
| --- | --- | --- | --- | --- | --- | --- | --- | --- | --- |
| 55a | Research | Luleå | 0,5 | 0,42 | 1,20 | 300 | 150 | - | - |
| 37a | Cloud, crypto | Boden | 30 |  | 1,03 | 7200 | 5000 | - | - |
| 7 | Colo | Esbogatan 5 | 16 | 11 | 1,15 | 6400 | 5600 | - | - |
| 31b | Colo, cloud | Kanslistvägen, Falkenberg | 3 | 3 | 1,54 | 4000 | 2500 | - | - |
| 38 | Colo, cloud | Boden | 25 | 20 | 1,07 | 4000 | - | - | - |
| 51 | Colo | Kista | 2 | 1,8 | - | 250 | 200 | - | - |
| 41 | Colo, cloud | Hudiksvall | 0,36 | 0,2 | 1,80 | 100 | 40 | - | - |
| 20 | Colo, cloud | Lidköping | 4 |  | - | 500 | 400 | - | - |
| 46 | Colo | Lund | 0,16 | 0,11 | - | 100 | 100 | - | - |
| - |  |  |  |  |  |  |  |  |  |
| 68 | Company, other | Kista |  |  |  |  |  |  |  |
| 4 | Company, other | Alingsås |  |  |  |  |  |  |  |
| 35b | Colo | Göteborg | 1,5 | 0,8 | 1,50 | 1500 | - | - | - |
| - | Colo, cloud | Akalla (Stockholm) | < 1 MW | < 1 MW | 1,30 | 470 | 470 | - | - |
| 17 | Colo | Upplands Väsby | 8 | 5 | 1,25 | 8000 | 6000 | - | - |

**Table A3.** Part two of the questionnaire. Specific technical data for those companies with a second data center.

| **Company** | **Type of DC** | **Municipality/adress** | **Ptot (MW)** | **Pit (MW)** | **PUE** | **Total surface area** | **Server area** | **H_2_O m^3^/år** | **WUE** |
| --- | --- | --- | --- | --- | --- | --- | --- | --- | --- |
| 55b | Research | - | 0,5 | 0,49 | 1,015 | 400 | 300 | - | - |
| 37b | Cloud | Robertsfors | 4 |  | 1,07 | 1500 | 1400 | - | - |
| 31a | Colo, cloud | Hägersten | 1 | 1 | 1,6 | 2400 | 1200 | 0 | - |
| 35a | Colo | Göteborg | 0,5 | 0,3 | 1,4 | 1000 | 500 |  |  |

Appendix B – Compilation data centers

Table to appendix B illustrates the estimated electricity use and environmental footprint for each DC. Regarding the promised confidentiality in the survey, no company names or exact positions are shown in the appendix. Enterprise names are exchanged for numbers, companies with more than one DC are also assigned a letter after the number.

**Table B1.** Illustrates information about the number of DCs at each geographic location. Together with data and calculations describing the DCs total power (*DC_P_total_*), electricity use (*DC_E_total_*), release of GHG emissions and water consumption.
* = Information obtained from the questionnaire survey.
** = Information obtained from the company's website or other web-based sources.
*** = Stencil data used from previous studies

| **Company** |  | **Municipality** | **Number of DCs** | **DC_P_total_ (MW)** | **DC_E_tot_ (GWh/år)** | **DC_GHG_ (tonCO2eq/år)** | **DC_indirect + direct H2O_ (m^3^/år)** |
| --- | --- | --- | --- | --- | --- | --- | --- |
| 1 | a | Helsingborg | 1 | 0,9 *** | 7,4 | 329 | 3,21E+04 |
| 1 | b | Malmö | 1 | 1,8 *** | 15,7 | 696 | 6,79E+04 |
| 2 | a | Stockholm | 1 | 1,8 *** | 16,2 | 298 | 6,53E+04 |
| 2 | b | Stockholm | 1 | 0,4 *** | 3,5 | 65 | 1,42E+04 |
| 2 | c | Stockholm | 1 |  | 0,0 |  |  |
| 3 |  | Karlskrona | 1 |  | 0,0 |  |  |
| 4 |  | Alingsås * | 1 |  |  |  |  |
| 5 | a | Katrineholm | 1 | 44 ** | 385,4 | 7102 | 1,56E+06 |
| 5 | b | Eskilstuna | 1 | 44 ** | 385,4 | 7102 | 1,56E+06 |
| 5 | c | Västerås | 1 | 44 ** | 385,4 | 7102 | 1,56E+06 |
| 6 |  | Umeå, Ersboda | 2 | 1,8 *** | 15,7 | 261 | 1,88E+05 |
| 7 |  | Stockholm | 1 | 16 * | 140,2 | 2583 | 5,65E+05 |
| 8 | a | Stockholm |  |  |  |  |  |
| 8 | b | Stockholm | 1 | 0,4 *** | 3,8 | 70 | 1,52E+04 |
| 8 | c | Stockholm | 1 | 1,15 ** | 10,1 | 186 | 4,06E+04 |
| 8 | d | Malmö | 1 | 0,5 ** | 4,4 | 194 | 1,90E+04 |
| 8 | e | Stockholm | 1 | 0,9 *** | 7,8 | 144 | 3,16E+04 |
| 8 | f | Stockholm | 1 | 3 ** | 26,3 | 484 | 1,06E+05 |
| 9 |  | Stockholm | 1 |  | 0,0 |  |  |
| 10 |  | Vallentuna | 1 | 3,2 *** | 27,6 | 508 | 1,11E+05 |
| 11 | a | Umeå | 1 |  |  |  |  |
| 11 | b | Stockholm | 1 |  |  |  |  |
| 11 | c | Malmö | 1 | 1,8 *** | 16,2 | 718 | 7,01E+04 |
| 12 |  | Gotland | 1 | 4,3 *** | 37,3 | 688 | 1,51E+05 |
| 13 | a | Sollentuna | 1 | 7,5 *** | 65,7 | 1211 | 2,65E+05 |
| 13 | b | Stockholm | 1 | 5,2 *** | 45,6 | 839 | 1,84E+05 |
| 14 | a | Stockholm | 1 |  |  |  |  |
| 14 | b | Österåker | 1 |  |  |  |  |
| 15 |  | Botkyrka | 1 |  |  |  |  |
| 16 | a | Eslöv | 1 |  |  |  |  |
| 16 | b | Ånge | 1 | 8,8 *** | 77,1 | 1285 | 9,25E+05 |
| 17 |  | Upplands Väsby * | 1 | 8 * | 70,1 | 1291 | 2,83E+05 |
| 18 |  | Boden | 1 | 2 *** | 17,2 | 303 | 2,11E+05 |
| 19 | a | Piteå | 1 | 1 ** | 8,8 | 154 | 1,07E+05 |
| 19 | b | Piteå | 1 | 1,8 ** | 15,8 | 277 | 1,93E+05 |
| 19 | c | Piteå (Hortlax) | 1 | 1,7 ** | 14,9 | 262 | 1,82E+05 |
| 19 | d | Stockholm | 2 | 4 ** | 35,0 | 646 | 1,41E+05 |
| 19 | e | Falun | 1 | 20 ** | 175,2 | 3228 | 7,07E+05 |
| 20 |  | Lidköping * | 1 | 4 * | 35,0 | 646 | 1,41E+05 |
| 21 | a | Stockholm | 1 | 3,2 *** | 28,0 | 517 | 1,13E+05 |
| 21 | b | Stockholm | 1 | 8 ** | 70,1 | 1291 | 2,83E+05 |
| 21 | c | Stockholm | 1 | 3,2 *** | 28,0 | 517 | 1,13E+05 |
| 22 | a | Sigtuna | 1 | 35,2 *** | 308,4 | 5682 | 1,24E+06 |
| 22 | b | Linköping | 1 | 22,9 *** | 200,4 | 3693 | 8,09E+05 |
| 23 |  | Jokkmokk | 1 | 8 ** | 70,1 | 1232 | 8,57E+05 |
| 24 |  | Luleå | 3 | 100 ** | 876,0 | 15404 | 1,07E+07 |
| 25 |  | Stockholm | 1 |  |  |  |  |
| 26 |  | Skellefteå |  |  |  |  |  |
| 27 |  | Helsingborg | 1 |  |  |  |  |
| 28 | a | Överkalix | 1 |  |  |  |  |
| 28 | b | Luleå | 1 |  |  |  |  |
| 28 | c | Älvsbyn | 1 |  |  |  |  |
| 29 |  | Lycksele | 1 | 0,5 *** | 4,0 | 67 | 4,85E+04 |
| 30 | a | Boden | 1 | 0,9 *** | 7,8 | 138 | 9,59E+04 |
| 30 | b | Boden |  |  |  |  |  |
| 31 | a | Stockholm | 1 | 1 * | 8,8 | 161 | 3,53E+04 |
| 31 | b | Falkenberg | 1 | 3 * | 26,3 | 1166 | 1,14E+05 |
| 32 | a | Malmö | 1 | 1 ** | 8,8 | 389 | 3,79E+04 |
| 32 | b | Göteborg | 1 | 1,3 ** | 11,4 | 210 | 4,59E+04 |
| 32 | c | Stockholm | 1 | 0,7 *** | 6,1 | 112 | 2,45E+04 |
| 32 | d | Stockholm | 1 | 1,6 ** | 14,0 | 258 | 5,65E+04 |
| 32 | e | Stockholm | 1 | 1,6 ** | 14,0 | 258 | 5,65E+04 |
| 32 | f | Stockholm | 1 | 5 ** | 43,8 | 807 | 1,77E+05 |
| 33 |  | Göteborg | 1 | 2,8 *** | 24,3 | 447 | 9,79E+04 |
| 34 |  | Avesta |  |  | 0,0 |  |  |
| 35 | a | Göteborg * | 1 | 0,5 * | 4,4 | 81 | 1,77E+04 |
| 35 | b | Göteborg * | 1 | 1,5 * | 13,1 | 242 | 5,30E+04 |
| 36 |  | Stockholm | 1 | 0,32 ** | 2,8 | 52 | 1,13E+04 |
| 37 | a | Boden * | 1 | 30 * | 262,8 | 4621 | 3,21E+06 |
| 37 | b | Robertsfors * | 1 | 4 * | 35,0 | 584 | 4,20E+05 |
| 38 |  | Boden * | 6 | 25 * | 219,0 | 3851 | 2,68E+06 |
| 39 |  | Kungälv | 1 | 4,4 *** | 38,5 | 710 | 1,56E+05 |
| 40 |  | Göteborg | 3 |  |  |  |  |
| 41 |  | Hudiksvall * | 1 | 0,36 * | 3,2 | 53 | 3,78E+04 |
| 42 | a | Stockholm | 5 | 11,5 ** | 100,7 | 1856 | 4,06E+05 |
| 42 | b | Stockholm | 1 | 5 ** | 43,8 | 807 | 1,77E+05 |
| 43 |  | Storuman | 1 |  |  |  |  |
| 44 |  | Landskrona | 1 |  |  |  |  |
| 45 |  | Jönköping | 1 |  |  |  |  |
| 46 |  | Lund * | 1 | 0,16 * | 1,4 | 62 | 6,07E+03 |
| 47 |  | Varberg | 1 |  |  |  |  |
| 48 | a | Gävle | 2 | 80 ** | 700,8 | 12914 | 2,83E+06 |
| 48 | b | Sandviken | 1 | 45 ** | 394,2 | 7264 | 1,59E+06 |
| 48 | c | Staffanstorp | 1 | 22 ** | 192,7 | 8549 | 8,35E+05 |
| 48 | d | Gävle | 1 | 40 ** | 350,4 | 6457 | 1,41E+06 |
| 49 |  | Stockholm | 1 |  |  |  |  |
| 50 | a | Stockholm | 1 |  |  |  |  |
| 50 | b | Trollhättan | 1 |  |  |  |  |
| 50 | c | Växjö | 1 |  |  |  |  |
| 50 | d | Lycksele | 1 |  |  |  |  |
| 51 |  | Stockholm * | 1 | 2 * | 17,5 | 323 | 7,07E+04 |
| 52 |  | Partille | 1 | 1,5 *** | 13,5 | 248 | 5,44E+04 |
| 53 |  | Stockholm | 1 |  |  |  |  |
| 54 |  | Västerås | 1 | 0,6 *** | 5,4 | 99 | 2,18E+04 |
| 55 | a | Luleå | 1 | 0,5 * | 4,4 | 77 | 5,35E+04 |
| 55 | b | Luleå | 1 | 0,5 * | 4,4 | 77 | 5,35E+04 |
| 56 |  | Åtvidaberg | 1 | 4,4 *** | 38,5 | 710 | 1,56E+05 |
| 57 |  | Vilhelmina | 1 | 8,5 *** | 74,5 | 1241 | 8,93E+05 |
| 58 | a | Borås | 1 | 0,4 *** | 3,4 | 62 | 1,36E+04 |
| 58 | b | Borås | 1 | 0,5 *** | 4,0 | 75 | 1,63E+04 |
| 59 |  | Lund/Veberöd | 2 |  |  |  |  |
| 60 |  |  | 1 | 2,1 *** | 18,7 | 828 | 8,09E+04 |
| 61 | a | Stockholm | 1 |  |  |  |  |
| 61 | b | Haninge | 1 |  |  |  |  |
| 61 | c | Stockholm | 1 |  |  |  |  |
| 61 | d | Solna | 1 | 1,3 *** | 11,5 | 211 | 4,62E+04 |
| 62 | a | Nacka | 1 | 1,8 *** | 16,2 | 298 | 6,53E+04 |
| 62 | b | Stockholm | 1 | 1,5 *** | 13,5 | 248 | 5,44E+04 |
| 63 |  | Älvsbyn | 1 | 9 *** | 78,4 | 1379 | 9,59E+05 |
| 64 |  | Stockholms län | 1 | 2,5 *** | 21,9 | 404 | 8,84E+04 |
| 65 |  | Gävle | 1 | 1,2 *** | 10,8 | 199 | 4,35E+04 |
| 66 |  | Göteborg | 1 |  |  |  |  |
| 67 |  | Luleå | 1 |  |  |  |  |
| 68 |  | Stockholm | 1 |  |  |  |  |
| 69 |  | Stockholm | 1 |  |  |  |  |
| 70 |  | Stockholm | 1 | 19 ** | 166,4 | 3067 | 6,71E+05 |
| 71 |  | Växjö | 1 | 0,6 *** | 5,5 | 243 | 2,38E+04 |
| **Totalt** |  |  |  | **761** | **6670** | **1,29E+05** | **4,16E+07** |
